# Pacing the phasing of leg and arm movements in breaststroke swimming to minimize intra-cyclic velocity fluctuations

**DOI:** 10.1371/journal.pone.0186160

**Published:** 2017-10-12

**Authors:** Josje van Houwelingen, Melvyn Roerdink, Alja V. Huibers, Lotte L. W. Evers, Peter J. Beek

**Affiliations:** 1 Department of Applied Physics, Eindhoven University of Technology and J.M. Burgers Centre for Fluid Dynamics, Postbus 513, 5600 MB Eindhoven, the Netherlands; 2 Department of Human Movement Sciences, Amsterdam Movement Science, Vrije Universiteit Amsterdam, van der Boechorststraat 9, 1081 BT Amsterdam, the Netherlands; University of California Merced, UNITED STATES

## Abstract

In swimming propelling efficiency is partly determined by intra-cyclic velocity fluctuations. The higher these fluctuations are at a given average swimming velocity, the less efficient is the propulsion. This study explored whether the leg-arm coordination (i.e. phase relation *ϕ*) within the breaststroke cycle can be influenced with acoustic pacing, and whether the so induced changes are accompanied by changes in intra-cyclic velocity fluctuations. Twenty-six participants were asked to couple their propulsive leg and arm movements to a double-tone metronome beat and to keep their average swimming velocity constant over trials. The metronome imposed five different phase relations *ϕ*_*i*_ (90, 135, 180, 225 and 270°) of leg-arm coordination. Swimmers adjusted their technique under the influence of the metronome, but failed to comply to the velocity requirement for *ϕ* = 90 and 135°. For imposed *ϕ* = 180, 225 and 270°, the intra-cyclic velocity fluctuations increased with increasing *ϕ*, while average swimming velocity did not differ. This suggests that acoustic pacing may be used to adjust *ϕ* and thereby performance of breaststroke swimming given the dependence of propelling efficiency on *ϕ*.

## Introduction

**Fig 1:**
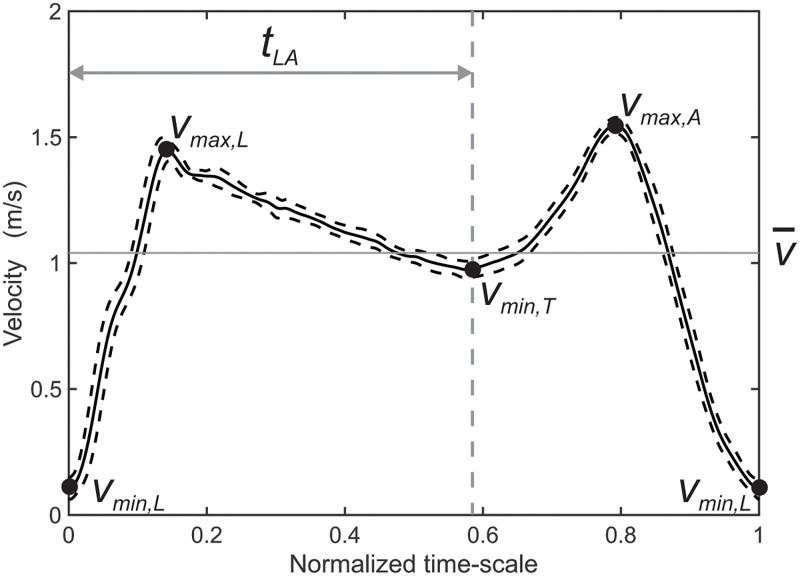
An exemplary velocity profile of a breaststroke cycle. The profile clearly exhibits intra-cyclic velocity variations, i.e. pronounced deviations from the average swimming velocity $\bar{v}$, as represented by the horizontal line. The characteristic points from Eqs 3 and 2 are indicated as local maxima and minima in the velocity profile. The profile was obtained by averaging 7 consecutive breaststroke cycles from one individual measured with automated LED tracking on a normalized time-scale. The standard deviation is indicated by the dashed lines, reflecting inter-cyclic variations. Note that in this trial the leg-arm phase coordination was around 210°.

Swimmers seek to improve their personal bests and set new records. Swimming more efficiently is one of the factors that may help to attain this goal. More efficient swimming implies converting a higher rate of power into forward speed, rather than into power losses. Efficiency may be studied in terms of the power balance:

$$\begin{array}{c} P_{s}=P_{d}+P_{p}=P_{\bar{v}}+P_{\Delta v}+P_{p} \end{array}$$ (1)

where *P*_*s*_ represents the power generated by the swimmer, *P*_*d*_ represents the power losses to drag forces, and *P*_*p*_ represents the power losses related a moving point of push-off (i.e. kinematic losses to water set in motion). *P*_*d*_ can be divided in a component related to the average speed of a swimmer $P_{\bar{v}}$ (effectively used power) and to a component related to the velocity fluctuations *P*_Δ*v*_ [1, 2]. In terms of *P*_*d*_ and drag force *F*_*d*_ (*P*_*d*_ = *F*_*d*_ ⋅ *v* ∼ *v*^2^ ⋅ *v*) [2], velocity fluctuations are inefficient and should be suppressed. However, the propulsive actions in a stroke inevitably lead to velocity fluctuations. Previous studies concluded that at a certain speed higher velocity fluctuations resulted in a higher energy expenditure of the swimmer [1, 3–6]. Due to the sequential character of the leg and arm movements in the breaststroke (and butterfly), the velocity fluctuations are comparatively higher and the efficiency is lower than in other stroke types [1, 3, 7]. In this study, the focus will be on the breaststroke, because the velocity fluctuations are comparatively high and because of the clear sequential nature of the stroke. This implies that there might be more opportunities for improving swimming efficiency by reducing the velocity fluctuations, by changing the coordination between the legs and the arms for instance. In Fig 1 a typical velocity profile of a breaststroke cycle is shown.

The velocity profile is bi-modal; the increase to the first local maximum is related to the propulsive phase of the legs and the increase to the second maximum is related to the propulsive phase of the arms. The strong reduction in speed is caused by the leg and arm recovery phase, which is accompanied by large drag forces. The slight decrease of the velocity after leg propulsion is associated with the glide phase of the stroke in which the swimmer extends the arms and legs [6, 8–11]. The velocity fluctuations of the breaststroke will be quantified by the intra-cyclic velocity variability *IVV* [4, 10, 11], given by:

$$\begin{array}{c} IVV=\frac{v_{max,L}-v_{min,L}+v_{max,A}-v_{max,T}}{\bar{v}}, \end{array}$$ (2)

with *v*_*max*, *L*_ representing the maximal velocity after the leg propulsion, *v*_*min*, *L*_ the minimal velocity before leg propulsion, *v*_*max*, *A*_ the maximal velocity after arm propulsion, *v*_*max*, *T*_ the local minimum in the velocity profile between leg and arm propulsion and $\bar{v}$ the mean velocity. These characteristic velocities are indicated in the velocity profile shown in Fig 1. Previous studies on breaststroke have shown that at a given speed higher *IVV* impose higher energy costs [4] and that the *IVV* was greater for low speed swimming conditions [11]. The *IVV* seem to be dependent on technique, since the *IVV* for undulating styles are smaller compared to a flat style, due to (de)acceleration of body parts above the water [12]. Moreover, it has been observed that the minimum hip velocity is higher for elite swimmers while the maximum velocity is similar, which suggests lower *IVV* [13]. However, it has also been found that the *IVV* of elite swimmers are higher, due to higher peak velocities needed to perform at higher speed and similar minimum velocities compared to non-elite swimmers [10].

The leg-arm coordination is an important performance determining factor. Previous studies have shown that the timing of the leg- and arm-propulsive phases, the glide phase and the recovery phase differs for different swimming levels [10, 13, 14] and events [9, 13]. For example, it has been reported that recreational swimmers do not employ a glide phase at all, while competitive swimmers can switch from a gliding coordination towards an overlapping coordination with increasing speed [11, 13, 14]. In this study the leg-arm phasing is quantified as the ratio of time spent between the onset of leg and arm propulsion *t*_*LA*_ and the total cycle time *t*_*tot*_:

$$\begin{array}{c} \phi =\frac{t_{LA}}{t_{tot}}\cdot 360{}^{\circ}. \end{array}$$ (3)

The *t*_*LA*_ is indicated in Fig 1, corresponding to a *ϕ* of ≈ 210°. It is assumed that the onset of leg and arm propulsion coincides with the local minima in the velocity profile [10] (see also the video in the supplementary material, S1 Movie).

It appears that there should be a relation between the relative phasing of the leg and arm movements and the *IVV*. It has already been suggested that optimizing the stroke cycle might be important for efficient breaststroke swimming [15]. The purpose of this study is to explore whether one’s preferred stroke cycle can be changed by a simple acoustic intervention and to examine the effects of the thus induced changes in coordination on the *IVV*. It is not yet evident which phasing between the legs and arms is optimal in breaststroke. Considering that low *IVV* are more efficient and that elite swimmers generally have lower *IVV* at specified speeds, determining the phase relation with the lowest *IVV* at a given speed, and training swimmers to coordinate their leg-arm coordination correspondingly, can help in optimizing the breaststroke performance. The modification of the phase relation of the leg and arm movements within the breaststroke might be achieved by a double-tone acoustic pacing signal. Inspired by more basic coordination dynamics studies examining acoustic pacing as a means to stabilize coordination [16, 17] and modulate the execution of cyclic movements [18–20], acoustic pacing for guiding cyclic movements has been employed to stabilize cadence in running and to effectively modulate stepping frequency and stepping phase of walking [21, 22]. Acoustic pacing in swimming has already been used as an accurate method to pace swim speed, in the sense that sounds at regular time intervals had to coincide with the swimmer progressing a certain distance [23]. It has also been used to constrain the stroke rate, in order to stabilize and slightly change the motor organization of swimmers in time to exhaustion tests [24, 25]. Furthermore, adding constraints (by instruction), for example about the glide duration, seemed a fruitful way to access the coordination adaptability. Finally, it has been concluded that the self-selected glide pattern showed less *IVV* and energy costs [26, 27]. Whether acoustic pacing can be used to modify the phase relation between leg and arm movements is still unknown.

The research questions for this study were thus twofold, namely:

- Can the phase relation between leg and arm movements within the breaststroke cycle be influenced using acoustic pacing while keeping the average swimming speed constant?
- And, if so, how do the imposed changes in the leg-arm coordination affect the intra-cyclic velocity variability?

## Method

### Participants

Twelve female and fourteen male swimmers, competing at the Dutch regional level or higher over at least the past four years, volunteered to participate in this study. Participants were 20.0 ± 3.3 years of age (mean ± standard deviation), 177.9 ± 8.5 cm tall, weighed 71.0 ± 10.6 kg, had 10.9 ± 3.8 years of experience and practised 5.4 ± 4.1 hours per week. Their personal bests on the 50 m breaststroke were 40.8 ± 3.8 s for the women and 35.1 ± 3.4 s for the men. All participants were healthy and had no injury, because this could have influenced swimming technique. Prior to the experiment, participants gave their written informed consent. The study was approved by the ethics committee of the Faculty of Behavioural and Movement Sciences of the Vrije Universiteit Amsterdam.

### Apparatus

The experiment was performed in the 50 m indoor training pool of the Tongelreep in Eindhoven, the Netherlands. During all trials, participants wore an underwater MP3-player (FINIS, Neptune) attached to their swimming goggle, transmitting the sound through bone conduction. Via this MP3-player a double-toned metronome beat was presented to prescribe the stroke frequency and the required phase relation (*ϕ*) between leg and arm movements. The beat for the onset of arm propulsion and onset of leg propulsion was identified by a high and low tone, respectively. The time interval between similarly pitched beeps was kept constant, corresponding to one’s preferred stroke frequency. However, the time interval between the high and low pitched beeps was varied to impose the following arm-leg phase relations: *ϕ* = 90, 135, 180, 225 and 270°. These double-toned metronome beeps were generated in Matlab (Mathworks release 2012a) for each individual swimmer.

**Fig 2:**
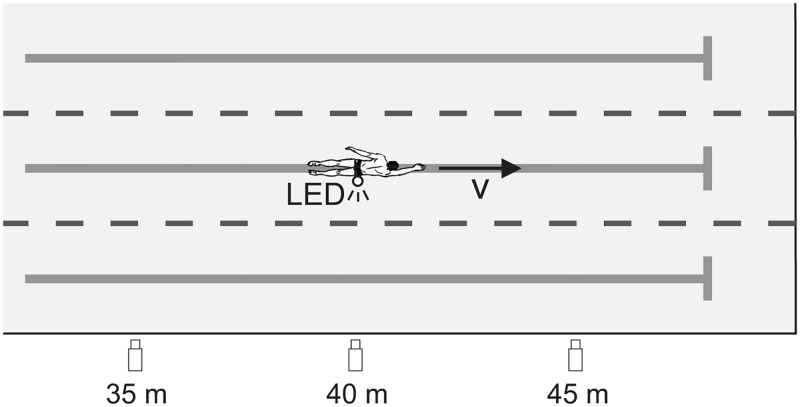
Setup for velocity tracking of the LED marker. Top view of the swimming pool with the synchronized cameras placed in the side wall of the pool. The cameras (at 35, 40 and 45 m) recorded the swimmer’s motion, using a LED marker on the hip.

Three cameras (Basler, sc1400gc, 50 fps, resolution: 788 × 524) positioned at 35, 40 and 45 m from the beginning of the lane, at a depth of 0.55 m in the side wall of the pool, were used for recording the swimmer’s motion in the sagittal plane (see Fig 2). The total recording range at the level of the swimmer (in the middle of the second lane from the side wall, ≈ 3.75 m) measured 17 m, which proved to be sufficient for capturing approximately seven swimming cycles.

A white LED light, placed at the swimmer’s hip, was used as a marker to track the horizontal and vertical position and instantaneous velocity of the swimmer. Although there is a difference between the velocity profiles of the hip and the true swimming velocity (measured at the center of gravity), the similarities for breaststroke are high and we therefore considered the hip velocity as a good estimate of the forward velocity profile [28, 29]. The position and velocity data were obtained using an automated tracking algorithm (in-house code in Matlab: developed by J. van Houwelingen, A.P.C. Holten (TU Eindhoven)). Sometimes there were indications of marker occlusions at the edge of the camera view. Due to overlapping views of the camera, the data were often not interrupted at relevant points. If data were interrupted, the affected stroke could be skipped during the analysis. Innosportlab de Tongelreep made available the calibration of the cameras for post-processing the data. On average 7 ± 2 stroke cycles were averaged to obtain the mean velocity profile. An example of an averaged velocity profile of a single participant obtained with this tracking algorithm was provided in Fig 1. As the application of the calibration required a selection of the vertical plane of interest parallel to the side of the pool (here chosen half-way the second lane at 3.75 m), deviations of the LED marker from this plane resulted in a small error in the velocity signal. However, since the *IVV* is a normalized quantity the effect on the final result is very limited.

A stopwatch (Elite sports) was used to measure lap times.

### Protocol

Prior to the experiment, participants did an individual warming up in the water. After the warm-up participants were instructed to swim 50 m breaststroke at maximal pace. 70% of this maximal velocity was determined as the exertion level for the remainder of the experiment, a pace that is commonly used in technique training. As a baseline test, participants were instructed to swim 50 m breaststroke at 70% of their maximal velocity. The preferred phase relation (*ϕ*_*p*_) and stroke frequency could be determined from this trial, as well as an indication for the lap time at the associated exertion level. The preferred stroke frequency was used for generating the metronome beats for the experimental trials. Next, the participants were instructed to practice the synchronization of the leg and arm movements to the metronome beats for two times 50 m (*ϕ*_*i*_ = 160 and 230°) at 70% of their maximal speed. Specifically, they were asked to synchronize their arm propulsion with the high tone and their leg propulsion with the low tone. These trials were excluded from the analysis.

For the experimental trials the participants were instructed to swim ten trials of 50 m breaststroke at a constant speed corresponding to 70% of the maximal velocity. Five different phase relations (*ϕ*_*i*_), ranging from 90 to 270° in increments of 45°, were imposed in randomized order. Each *ϕ*_*i*_ was imposed twice in succession. The first trial associated with each pacing regime experienced was treated as a practice trial, which was not used in the analysis. The participants did not receive prior information about the *ϕ*_*i*_ during the trials. In the end, the participants were instructed to swim a final trial at 70% of their maximal velocity, without acoustic pacing.

Directly after each trial, the participants were asked to indicate how fatigued they felt, i.e. rate of perceived exertion (*RPE*), and how difficult the imposed phase relation (*D*_*ϕ*_) was, both assessed using a Borg scale [30]. Between trials they rested for a couple of minutes while they were informed about their lap time.

### Data analysis

After capturing the position data in pixels for each camera using the tracking algorithm (see supplementary material for an exemplary video, S1 Movie), the data were converted to world coordinates in meters (m) using the calibration, differentiated to obtain the instantaneous velocity (in m/s) and combined into a single velocity array. Single stroke cycles were selected by manually selecting the area of characteristic peaks within the velocity profile. Peaks were then automatically picked using the local minimum. Stroke cycles were time-normalized in order to obtain a mean velocity profile per trial. The phase relation *ϕ* and the intra-cyclic velocity fluctuations *IVV* were determined from this mean velocity profile, by determining the characteristic points as indicated in Eqs (3) and (2) similar to the peak picking criteria for single stroke cycles. The intra-individual standard deviation *σ*_*ϕ*_ was determined by taking the standard deviation over the *ϕ* over all strokes in a trial. The average velocity $\bar{v}$ was calculated based on the position and time of the start of the first stroke cycle and finish of the last stroke within the camera view.

### Statistical analysis

The effect of imposed phasing (5 levels: *ϕ*_*i*_ = 90, 135, 180, 225, 270°) on the mean executed phase relation (${\bar{\phi }}_{e}$), the intra-individual standard deviation of the executed phase relation over the included swimming cycles (*σ*_*ϕ*_), intra-cyclic velocity fluctuations (*IVV*), mean swimming velocity ($\bar{v}$), rate of perceived exertion (*RPE*) and rate of perceive difficulty (*D*_*ϕ*_) was examined. All statistical tests were conducted in IBM SPSS statistics 23. Values are given as (inter-individual) mean ± standard deviation. The effects were considered significant at *p* < 0.05. The ${\bar{\phi }}_{e}$, *σ*_*ϕ*_, *IVV* and $\bar{v}$ were examined using a 1 × 5 analysis of variance (ANOVA) with repeated measures. Effect sizes are represented as partial eta squared values ($\eta_{p}^{2}$) and were considered low, moderate and high for $\eta_{p}^{2}∼0.02,0.13$ and 0.26 respectively. When the sphericity assumption was violated, the degrees of freedom were adjusted using the Greenhouse-Geisser adjustment (if *ϵ* < 0.75) or the Huyn-Feldt adjustment (if *ϵ* > 0.75). For analysing the pairwise comparison within different conditions a Bonferroni adjustment was applied. Because of their ordinal scale, the subjective ratings of the Borg scales (score 6–20) were examined using a non-parametric Friedman test. When the Borg scales varied significantly over the conditions, Wilcoxon-signed-rank tests were conducted to compare differences between conditions. A Bonferroni correction was applied to correct for the number of tests and for potential false-negative outcomes (*p* < 0.005). The outcomes of unpaced trials before and after the block of paced trials were compared with dependent *t*-tests.

## Results

In Table 1 the statistical effects of the dependent variables are summarized.

**Table 1:** Summary of the statistical results. On the left side the results of the ANOVAs and on the right side the results of the non-parametric Friedman tests executed on the dependent variables as function of the imposed phase relation *ϕ*_*i*_.

Dependent variable	*F*	*p*	$\eta_{p}^{2}$		*χ*^2^	*p*
${\bar{\phi }}_{e}$	*F*(1.857, 46.431) = 52.989	¡0.001	0.679	*RPE*	*χ*^2^(4) = 26.579	¡0.001
*σ*_*ϕ*_	*F*(2.670, 66.749) = 3.985	< 0.05	0.137	*D*_*ϕ*_	*χ*^2^(4) = 48.685	¡0.001
$\bar{v}$	*F*(2.457, 61.435) = 25.461	¡0.001	0.505			
*IVV*	*F*(1.993, 49.832) = 16.902	¡0.001	0.403			

*abbreviations*: ${\bar{\phi }}_{e}$ = mean executed phase relation *ϕ*_*e*_, *σ*_*ϕ*_ = intra-individual standard deviation of *ϕ*_*e*_, $\bar{v}$ = mean velocity, *IVV* = intra-cyclic velocity fluctuations, *RPE* = rate of perceived exertion, *D*_*ϕ*_ = rate of perceived difficulty

In Table 2 the results of the comparison of the baseline and final trial are summarized.

**Table 2:** Results of the unpaced trials. Mean values and standard deviations, as well as the outcome of the dependent *t*-test of the baseline and final trial are given.

Dependent variable	Baseline	Final	*t*	*p*
${\bar{\phi }}_{p}$	205.2 ± 27.0	195.9 ± 23.2	*t*(25) = 4.065	¡0.001
$\bar{v}$	0.94 ± 0.08	0.93 ± 0.10	*t*(25) = 1.008	0.323
*IVV*	1.68 ± 0.19	1.70 ± 0.21	*t*(25) = −0.968	0.342
*RPE*	9.50 ± 1.50	9.69 ± 2.22	*t*(25) = −0.680	0.503

*abbreviations*: ${\bar{\phi }}_{p}$ = mean preferred phase relation *ϕ*_*r*_, $\bar{v}$ = mean velocity, *IVV* = intra-cyclic velocity fluctuations, *RPE* = rate of perceived exertion

All dependent variables were significantly affected by the imposed phase relation *ϕ*_*i*_ as shown in Table 1. The results of the dependent variables are presented later in this section.

**Fig 3:**
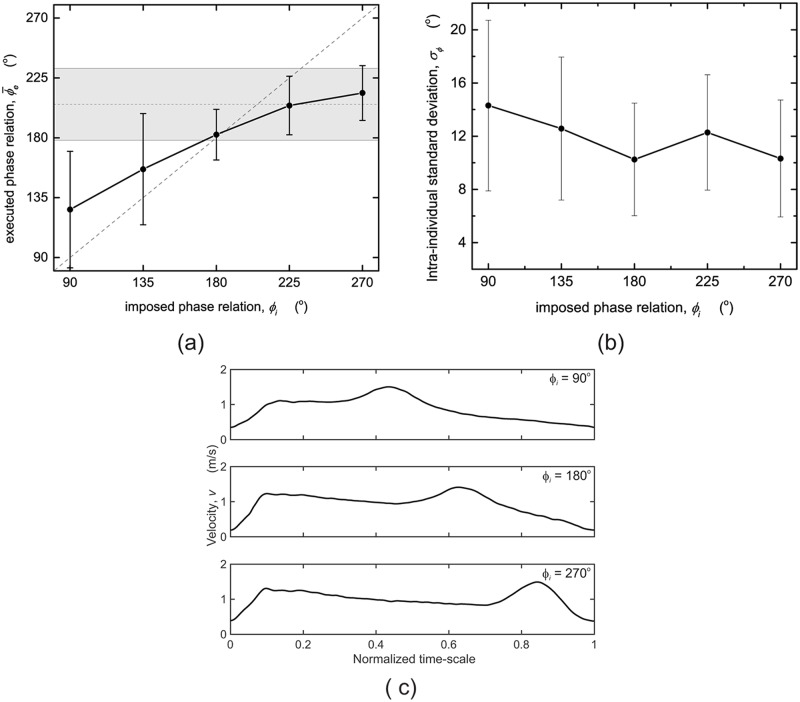
Executed phase relation *ϕ*_*e*_. (a) Mean ${\bar{\phi }}_{e}$ as a function of the imposed phase relation *ϕ*_*i*_. The grey dashed lines are the line of identity and the mean preferred phase relation ${\bar{\phi }}_{p}$ respectively. The shaded area represents the standard deviation from the ${\bar{\phi }}_{p}$. (b) Intra-individual standard deviation *σ*_*ϕ*_ as a function of *ϕ*_*i*_. Standard deviations are indicated by the vertical bars. (c) Typical mean velocity profiles for three different *ϕ*_*i*_ obtained from one participant.

In Fig 3a the means of the executed phase relation ${\bar{\phi }}_{e}$ per *ϕ*_*i*_ are shown. The dashed horizontal line with shaded area shows the mean preferred phase relation ${\bar{\phi }}_{p}=205.2\pm 27.0{}^{\circ}$ resulting from the unpaced baseline trial. The dashed diagonal represents the line of identity, where *ϕ*_*e*_ equals *ϕ*_*i*_. To illustrate the effect of imposing phase relations with acoustic pacing, Fig 3c shows the mean velocity profile of a participant for three conditions. The velocity profile is clearly affected by *ϕ*_*i*_, and therewith *t*_*LA*_ and *ϕ*_*e*_.

A post-hoc test showed all ${\bar{\phi }}_{e}$’s differed significantly from each other, indicating a significant increase with *ϕ*_*i*_. As can be appreciated from Fig 3a, the participants’ leg-arm coordination deviated from the imposed phase relations for all but *ϕ*_*i*_ = 180°, where ${\bar{\phi }}_{e}$ was 182.4 ± 19.1°, with larger deviations for *ϕ*_*i*_ further away from 180°. Post-hoc analyses for the intra-individual standard deviation *σ*_*ϕ*_ (a measure for the variation of *ϕ*_*e*_ over stroke cycles of an individual, see Fig 3b) revealed that *σ*_*ϕ*_ was significantly larger for *ϕ*_*i*_ = 90° (14.3°, *p* < 0.05) compared to *ϕ*_*i*_ = 180°. Paired-samples *t*-tests for the two unpaced conditions showed that the preferred phase relation ${\bar{\phi }}_{p}$ of the baseline trial (205.2 ± 27.0°) differed significantly from that of the final trial (195.9 ± 23.2°: *t*(25) = 4.065, *p* = 0.000).

**Fig 4:**
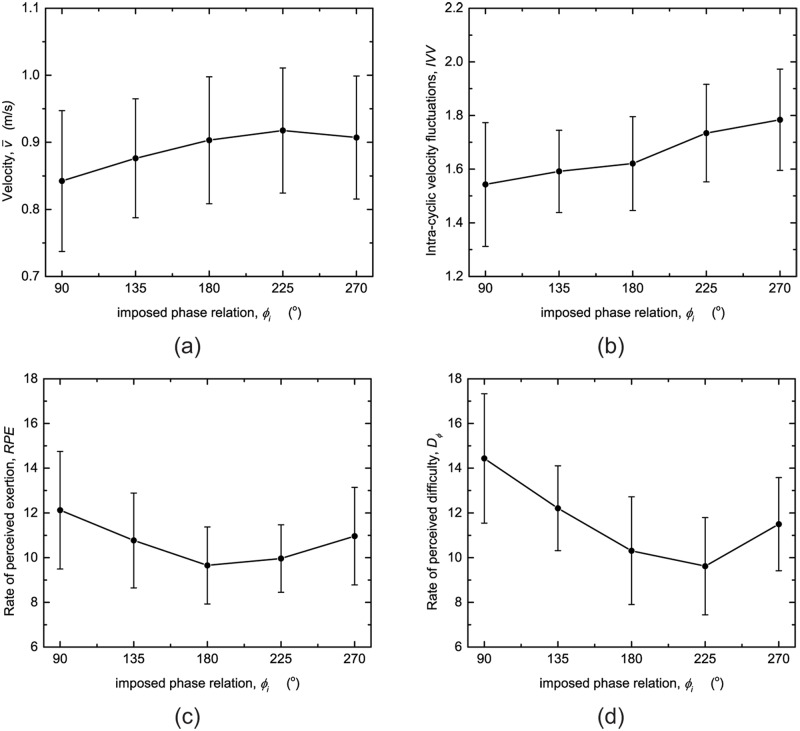
Effects of imposed phase relation *ϕ*_*i*_ on mean velocity $\bar{v}$ (a), intra-cyclic velocity fluctuations *IVV* (b) rate of perceived exertion *RPE* (c) and rate of perceived difficulty *D*_*ϕ*_ (d). Vertical bars represent the standard deviation.

Fig 4a shows the results of the mean velocity $\bar{v}$ per *ϕ*_*i*_. In the conditions with *ϕ*_*i*_ = 90° and 135° $\bar{v}$ was significantly lower compared to the mean velocities in the other conditions *p* < 0.01 and *p* < 0.05, respectively, which were not significantly different from each other.

In Fig 4b the results of the mean *IVV* are depicted. Post-hoc tests showed that *IVV* for *ϕ*_*i*_ = 225 and 270° were significantly higher than *ϕ*_*i*_ = 90° (*p* < 0.05 and *p* < 0.01, respectively), *ϕ*_*i*_ = 135° (*p* < 0.01 and *p* = 0.000, respectively) and *ϕ*_*i*_ = 180° (*p* = 0.000 and *p* = 0.000, respectively). All other pair-wise comparisons were not significant.

In Fig 4c the results of the rate of perceived exertion *RPE*, expressed in a mean Borg score, are shown. The post-hoc test showed that the *RPE* for the *ϕ*_*i*_ = 90° condition compared to 180° (*p* < 0.005) and 90° compared to 225° (*p* < 0.005) were significantly different from each other. Overall, the participants found the condition *ϕ*_*i*_ = 90° (12.12) the most exerting and the conditions *ϕ*_*i*_ = 180° (9.65) and 225° (9.96) the least exerting.

Lastly, the results of the rate of perceived difficulty *D*_*ϕ*_, expressed in a mean Borg score, are depicted in Fig 4d. Following the result of the post-hoc test, *D*_*ϕ*_ for all conditions were significantly different from each other (*p* < 0.005), except for the comparisons between the conditions *ϕ*_*i*_ = 135° and 270°, between 180° and 225° and between 180° and 270°, indicative of a parabolic relation with a minimum close to *ϕ*_*p*_. The participants found *ϕ*_*i*_ = 90° significantly more difficult (14.44) to perform than all other *ϕ*_*i*_.

## Discussion

The aim of this study was twofold. First of all, it was determined whether the participants were able to adjust their leg-arm coordination to acoustically imposed phase relations *ϕ*_*i*_. Based on the results (Table 1 and Fig 3), it can be concluded that the executed phase relations ${\bar{\phi }}_{e}$ were significantly affected by acoustic pacing, and that ${\bar{\phi }}_{e}$ increased with increasing *ϕ*_*i*_. Participants adhered to the *ϕ*_*i*_ = 180°, which was close to participants’ self-selected leg-arm coordination *ϕ*_*p*_, as observed in the two unpaced trials.

However, participants did not succeed in reproducing imposed leg-arm coordinations other than 180°, presumably due to a competition between following the imposed pattern and maintaining the participant’s preferred leg-arm coordination. Since the latter type of coordination stems from the interplay between neural (e.g., different central pattern generators) and biomechanical constraints (e.g., different eigenfrequencies of leg and arm movements), it is understandable that such a competition may occur, especially when large deviations are imposed. This was indeed the case for *ϕ*_*i*_ = 90°, with an imposed pattern deviating most from the participant’s self-selected leg-arm coordination, for which *σ*_*ϕ*_ was significantly larger suggesting less stable leg-arm coordination. Correspondingly, the participants’ rating of perceived difficulty *D*_*ϕ*_ (Fig 4d) was significantly higher for *ϕ*_*i*_ = 90° than for all other conditions.

The second aim was to examine if imposed changes in leg-arm coordination affected intra-cyclic velocity fluctuations (*IVV*). Since *IVV* is dependent on the average swimming velocity $\bar{v}$ [11], it is imperative for a proper comparison of the data that $\bar{v}$ remains constant across conditions. This requirement was not met for *ϕ*_*i*_ = 90 and 135°, where $\bar{v}$ was significantly lower than in the three other conditions (Fig 4a). Therefore, the interpretation of the effect of *ϕ* on *IVV* will be restricted to the *ϕ*_*i*_ = 180, 225 and 270° conditions. For these conditions, the *IVV* for *ϕ*_*i*_ = 180° was significantly lower than those for *ϕ*_*i*_ = 225 and 270° (Fig 4b). Based on the theoretical assumption that propelling efficiency might be enhanced by reducing *IVV* at a given swimming velocity, it can be concluded that swimming with a *ϕ* around 180° is optimal (i.e. among the examined conditions complying to the velocity requirement).

Note that in the present study the most efficient imposed phase relation was close to the participants’ self-selected *ϕ*_*p*_ observed in the unpaced trials. This is consistent with the findings of Seifert et al. [26, 27], who observed that the self-selected glide pattern resulted in the lowest *IVV*. Does this imply that our participants already attained the most efficient leg-arm coordination? Although the results of the ratings of perceived exertion and difficulty (Fig 4) point in that direction, the significant difference in self-selected leg-arm coordination between the baseline and final unpaced trials is worth mentioning. That is, at baseline, participants’ self-selected leg-arm coordination was 205.2°. After executing different acoustically imposed leg-arm coordination patterns, participants changed their self-selected leg-arm coordination to 195.9°, that is, closer to the *ϕ*_*i*_ = 180° for which we observed the lowest *IVV*. However, no significant effect was observed for $\bar{v}$, *IVV* and *RPE*) (Table 2). This immediate learning effect may be related to the concept of differential learning [31]. According to this notion, motor learning benefits from invoking larger than usual variations in task execution during practice allowing the actor to experience the differences among operating in different parts of the coordinative work space in order to help discover optimal solutions. Such variations may be brought about through task instructions or by manipulating organismic and/or environmental constraints (e.g., fatigue, or the surface or medium). Likewise, acoustic pacing of the leg-arm phasing may be exploited to expose the swimmer to different leg-arm coordination patterns, providing essential information to a swimmer about the manner in which the breaststroke can be performed best. Given the emphasis on drills in current swim training, it would be interesting to explore this possibility in greater detail in future work, along with imposed variations in frequency and speed.

In this study, a fairly wide range of *ϕ* was explored. For a follow-up study it is recommended to vary *ϕ*_*i*_ in the direct vicinity of *ϕ*_*p*_ and to use smaller increments of *ϕ*_*i*_ to determine the optimal *ϕ*. Moreover, it is recommended to pace the velocity (using visual feedback) to gain a better control over the velocity throughout the different trials. In the present study the swimmers were examined while swimming at 70% of their maximal velocity, a pace that is commonly used in technique training. Note that *IVV* are highly dependent on *v* [10, 11]. In contrast to previous studies [11], we found that the *IVV* were lower for slower velocities (in the *ϕ*_*i*_ = 90 and 135° conditions). This might be explained by the particulars of our study, in which a technique variation was acoustically imposed, whereas previous studies allowed participants to swim at different velocities without stroke constraints. It would be of interest to examine the effects of acoustic pacing on propelling efficiency when swimming at different paces (e.g. race pace). The same holds for the level of expertise of the swimmer.

The velocity profile is a result of the propulsive actions of the legs and arms. Throughout this study it is assumed that the onset of the propulsive actions of the legs and arms coincides with the minima indicated in Fig 1, on the basis of which we determined the executed leg-arm coordination (Eq 2). We did not quantify auditory-motor coordination [21], as this requires knowledge about beat onsets in relation to the velocity profile, which was not available since the measurement devices were not synchronized. Nevertheless, the key finding of our study stands, i.e. leg-arm coordination and thereby *IVV* can be readily modulated using acoustic pacing.

## Conclusion

Within certain margins, breaststroke swimmers can adjust the phase relation between their leg and arm movements to an acoustically prescribed phase relation *ϕ*. It was not possible to draw a clear-cut conclusion about the optimal *ϕ* regarding intra-cyclic velocity fluctuations, because the average velocity varied significantly over pacing conditions, with slower velocities for imposed phase relations of 90 and 135°. The average swimming velocity did not differ for imposed phase relations of 180, 225 and 270°, with the former being the most efficient coordination among the studied patterns, yielding significantly lower *IVV*. Exposure to different leg-arm coordination with pacing resulted in a change in participants self-selected leg-arm coordination towards the most efficient pattern in a subsequent unpaced condition, suggesting that pacing the phasing may be a fruitful entry point for coaches in discovering optimal swimming techniques.

## Supporting information

S1 Movie An exemplary movie of the automated tracking algorithm. (MP4)

S1 File Datasheet used for the statistical analysis. (SAV)

S1 Archive Zip-file with tracking data. (ZIP)
